# 1908. Social Risk Factors for COVID-19-Related Hospitalizations in Adults

**DOI:** 10.1093/ofid/ofac492.1535

**Published:** 2022-12-15

**Authors:** Olivia D Reese, Ashley Tippett, Laila Hussaini, Luis W Salazar, Meg Taylor, Caroline R Ciric, Khalel De Castro, Grace Taylor, Chris Choi, Laura A Puzniak, Robin Hubler, Srinivas Valluri, Benjamin Lopman, Satoshi Kamidani, Christina A Rostad, John M McLaughlin, Evan J Anderson

**Affiliations:** Emory University, Atlanta, Georgia; Emory University, Atlanta, Georgia; Emory Univeristy, Atlanta, Georgia; Emory University, Atlanta, Georgia; Emory University, Atlanta, Georgia; Emory University, Atlanta, Georgia; Emory University, Atlanta, Georgia; Emory University, Atlanta, Georgia; Emory University, Atlanta, Georgia; Pfizer Inc., Collegeville, Pennsylvania; Pfizer Inc., Collegeville, Pennsylvania; Pfizer Inc, New York, New York; Rollins School of Public Health | Emory University, Atlanta, Georgia; Emory University School of Medicine and Children's Healthcare of Atlanta, Atlanta, Georgia; Emory University School of Medicine and Children's Healthcare of Atlanta, Atlanta, Georgia; Pfizer, Collegeville, Pennsylvania; Emory University School of Medicine, Atlanta, Georgia

## Abstract

**Background:**

During the COVID-19 pandemic, social interventions such as social distancing and mask wearing have been encouraged. Social risk factors for SARS-CoV-2 infection and subsequent hospitalization remain uncertain.

**Methods:**

Adult patients were eligible if admitted to Emory University Hospital or Emory University Hospital Midtown with acute respiratory infection (ARI) symptoms (≤ 14 days) or an admitting ARI diagnosis from May 2021 – Feb 2022. After enrollment, an in-depth interview identified demographic and social factors (e.g., employment status, smoking history, alcohol use), household characteristics, and pandemic social behaviors. All patients were tested for SARS-CoV-2 using PCR. We evaluated whether these demographic and social factors were related to a positive SARS-CoV-2 test upon admission to hospital with ARI using a logistic regression model.

**Results:**

1141 subjects were enrolled and had SARS-CoV-2 PCR results available (700 positive and 441 negative). The median age was greater in the SARS-CoV-2 negative cohort than in the positive cohort (60 and 53 years, respectively; P< .0001). Those who tested positive were more likely to have had at least some college education compared to those who tested negative (64.3% vs 52.3%, P< .0001; adjusted odds ratio [aOR]: 1.4 [95%CI: 1.1, 2.0]). Compared to those who tested negative, those who were SARS-CoV-2 positive were also more likely to be employed (48.9% vs 26.5%, P< .0001; aOR: 1.7 [95%CI: 1.1, 2.3]), have children 5-17 yo at home (27.6% vs 17.9%, P=.0002; aOR: 1.5 [95%CI: 1.1, 2.1]). Those with COVID-19 were less likely to receive home healthcare (6.2% vs 13.3%, P< .0001; aOR: 0.5 [95%CI: 0.4, 0.9]) and to be a current or previous smoker (7.6% vs 17.7%, P< .0001; aOR: 0.3 [95%CI: 0.2, 0.5]).

**Conclusion:**

Among adults admitted to the hospital for ARI, those who tested positive for SARS-CoV-2 were typically younger, more likely to care for school-aged children, more likely to work outside the home, but were less likely to receive home healthcare or smoke. Personal and public health strategies to mitigate COVID-19 should take into consideration modifiable social risk factors.

**Disclosures:**

**Laura A. Puzniak, PhD. MPH**, Merck & Co., Inc.: Stocks/Bonds|Pfizer, Inc.: Stocks/Bonds **Robin Hubler, MS**, Pfizer Inc.: Employee|Pfizer Inc.: Stocks/Bonds **Srinivas Valluri, PhD**, Pfizer: Employee|Pfizer: Stocks/Bonds **Benjamin Lopman, PhD**, Epidemiological Research and Methods, LLC: Advisor/Consultant **Satoshi Kamidani, MD**, NIH: His institution (Emory University) receives funds from Pfizer for his work as a co-investigator on clinical trials of Pfizer COVID-19 vaccine.|Pfizer: His institution (Emory University) receives funds from Pfizer for his work as a co-investigator on clinical trials of Pfizer COVID-19 vaccine. **Christina A. Rostad, MD**, BioFire Inc, GSK, MedImmune, Micron, Merck, Novavax, PaxVax, Pfizer, Regeneron, Sanofi-Pasteur.: Grant/Research Support|Meissa Vaccines, Inc.: Co-inventor of RSV vaccine technology licensed to Meissa Vaccines, Inc.|NIH (Funding from NIH to conduct clinical trials of Moderna and Janssen COVID-19 vaccines): Grant/Research Support **John M. McLaughlin, PhD**, Pfizer: Employee|Pfizer: Stocks/Bonds **Evan J. Anderson, MD**, GSK: Advisor/Consultant|GSK: Grant/Research Support|Janssen: Advisor/Consultant|Janssen: Grant/Research Support|Kentucky Bioprocessing, Inc: Data Safety Monitoring Board|MedImmune: Grant/Research Support|Medscape: Advisor/Consultant|Merck: Grant/Research Support|Micron: Grant/Research Support|NIH: Funding from NIH to conduct clinical trials of Moderna and Janssen COVID-19 vaccines|PaxVax: Grant/Research Support|Pfizer: Advisor/Consultant|Pfizer: Grant/Research Support|Regeneron: Grant/Research Support|Sanofi Pasteur: Advisor/Consultant|Sanofi Pasteur: Grant/Research Support|Sanofi Pasteur: Data Adjudication and Data Safety Monitoring Boards|WCG and ACI Clinical: Data Adjudication Board.

